# Patterns and Drivers of Surface Energy Flux in the Alpine Meadow Ecosystem in the Qilian Mountains, Northwest China

**DOI:** 10.3390/plants14020155

**Published:** 2025-01-07

**Authors:** Yongxin Tian, Zhangwen Liu, Yanwei Fan, Yongyuan Li, Hu Tao, Chuntan Han, Xinmao Ao, Rensheng Chen

**Affiliations:** 1 College of Energy and Power Engineering, Lanzhou University of Technology, Lanzhou 730050, China; 17793527127@163.com (Y.T.); fanyanwei24@163.com (Y.F.); 2 Key Laboratory of Ecological Safety and Sustainable Development in Arid Lands, Northwest Institute of Eco-Environment and Resources, Chinese Academy of Sciences, Lanzhou 730000, China; hancht@lzb.ac.cn (C.H.); aoxinmao23@mails.ucas.ac.cn (X.A.); crs2008@lzb.ac.cn (R.C.); 3 Qilian Forestry and Grassland Administration, Haibei 810200, China; owen1231@sina.com (Y.L.); betty1231@163.com (H.T.); 4 University of Chinese Academy of Sciences, Beijing 100049, China

**Keywords:** alpine meadow, radiation balance, energy flux, energy partitioning

## Abstract

Alpine meadows are vital ecosystems on the Qinghai–Tibet Plateau, significantly contributing to water conservation and climate regulation. This study examines the energy flux patterns and their driving factors in the alpine meadows of the Qilian Mountains, focusing on how the meteorological variables of net radiation (*R_n_*), air temperature, vapor pressure deficit (*VPD*), wind speed (*U*), and soil water content (*SWC*) influence sensible heat flux (*H*) and latent heat flux (*LE*). Using the Bowen ratio energy balance method, we monitored energy changes during the growing and non-growing seasons from 2022 to 2023. The annual average daily *R_n_* was 85.29 W m^−2^, with *H*, *LE*, and *G* accounting for 0.56, 0.71, and −0.32 of *R_n_*, respectively. Results show that *R_n_* is the main driver of both *H* and *LE*, highlighting its crucial role in turbulent flux variations. Additionally, a negative correlation was found between air temperature and *H*, suggesting that high temperatures may suppress *H*. A significant positive correlation was observed between soil moisture and *LE*, further indicating that moist soil conditions enhance *LE*. In conclusion, this study demonstrates the impact of climate change on energy distribution in alpine meadows and calls for further research on the ecosystem’s dynamic responses to changing climate conditions.

## 1. Introduction

Alpine meadows are widely distributed across the Qinghai–Tibet Plateau (QTP), covering an area of approximately 700,000 km^2^, and represent a dominant vegetation type in this region [1,2]. Its unique growth environment is characterized by high altitude, low temperatures, intense radiation, short growth periods, low rainfall, and strong winds, making it more sensitive and vulnerable to climate change than other grassland ecosystems [3,4,5,6,7,8]. Studies have shown that surface temperatures in the region have significantly increased over the past few decades, at a rate twice the global average, affecting the structure and function of alpine meadow ecosystems [9,10]. Some alpine meadows have degraded, leading to rising surface temperatures and increased heat flux, which in turn alters energy distribution patterns and climate feedback mechanisms [11,12,13,14].

The energy exchange between the land and atmosphere is fundamental to ecosystem function and is a key process for surface ecosystems [15,16,17]. Changes in this process are influenced by meteorological factors, vegetation, and soil moisture, and serve as drivers of climate change. Most surface flux studies on the QTP focus on the growing season (GS), with some extending year-round. The energy distribution patterns of vegetation in alpine regions vary significantly between the GS and non-growing season (NGS) [4,18,19,20,21,22]. Extensive energy monitoring experiments across different regions of the QTP have shown that, in spatial patterns, *LE* is the main heat flux between the surface and atmosphere in the eastern QTP [23,24,25]. In terms of temporal patterns, some studies indicate that energy fluxes vary by season, with *H* dominating in the NGS and *LE* dominating in the GS [26]. Other studies have found that since the beginning of the 21st century, the overall trends of *H* and *LE* across the plateau have been increasing [27].

Currently, the eddy correlation method and the Bowen ratio energy balance (BREB) method are the primary tools for monitoring energy turbulence changes on the QTP [28,29,30,31]. However, research on the relationship between environmental controls and the energy balance remains insufficient [19,32,33]. Studies suggest that surface flux changes in the QTP are significantly influenced by the Asian monsoon [34]. Precipitation increases soil moisture, which affects climate change, enhances vegetation and soil evaporation, lowers soil temperatures, and thus reduces *H* [35]. *R_n_* is a crucial driving factor for the growth of surface vegetation; its value varies with vegetation type and determines surface temperature, influencing the temperature for vegetation growth [4,36]. An increase in *R_n_* and a decrease in precipitation will inhibit photosynthesis in vegetation, while varying *VPD* across different periods determines changes in *LE*; elevated *VPD* and *T_a_* will intensify leaf evaporation, thereby increasing *LE* [4,37]. In addition to accurately monitoring changes in energy turbulence and distribution patterns, it is also essential to reveal the key environmental factors that influence energy processes. The BREB is a simple and low-cost micrometeorological method for calculating surface energy fluxes and is currently a reliable approach for studying the relationships between vegetation, surface fluxes, and evaporation in different ecological environments [38,39,40].

To understand the patterns of surface energy flux and climate change during different periods of growth in the alpine meadow ecosystem [41], we monitored ecological factor data on vegetation changes in the alpine meadows of the northeastern QTP, specifically in the Qilian Mountains, across different growth stages. By studying the relationships between surface energy changes, distribution patterns, and climate change control factors, this research aims to achieve the following: (1) to identify the seasonal and diurnal patterns of energy exchange between alpine meadows and the atmosphere; (2) to investigate the driving factors of energy distribution in alpine meadow ecosystems influenced by climate and environmental elements.

## 2. Results

### 2.1. The Environmental Variables

**Figure 1:**
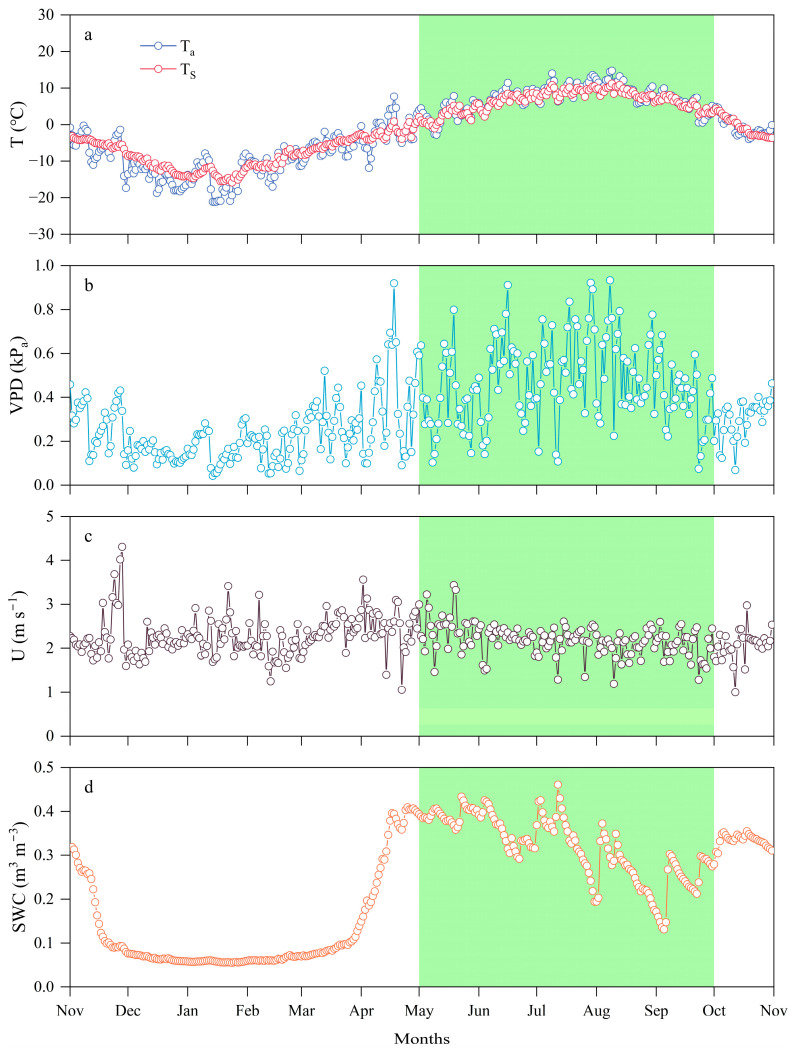
Annual variation in daily average meteorological variables at the meteorological observation site from 2022 to 2023, with the green area representing the GS: (**a**) *T_a_* and *T_s_*; (**b**) *VPD*; (**c**) *U*; (**d**) *SWC*.

Figure 1 shows the variation trends of environmental factors (*T_a_*, *T_s_*, *VPD*, *U*, and *SWC*) in the alpine meadow from 2022 to 2023, with specific data provided in Table 1. Throughout the vegetation growth period, the trends of *T_a_* and *T_s_* were similar, both reaching their minimum in January during the NGS, gradually increasing, peaking in July and August during the GS, and then gradually decreasing. During the cold NGS, *VPD* also decreases, reaching its minimum daily average in January, then gradually increasing and peaking during the warm and humid GS, with noticeable fluctuations in daily *VPD*. In contrast, *U* exhibits greater daily fluctuations during the NGS, whereas it remains more stable with smaller variations during the GS. The *SWC* is significantly influenced by air temperature throughout the vegetation growth period: It sharply declines starting in November, and from December to March, due to soil freezing, the daily average *SWC* remains low and stable. Starting in April, *SWC* rises rapidly, peaking during the GS, and then decreases again at the end of the GS.

### 2.2. Daily Variations in the Characteristics of Energy

**Figure 2:**
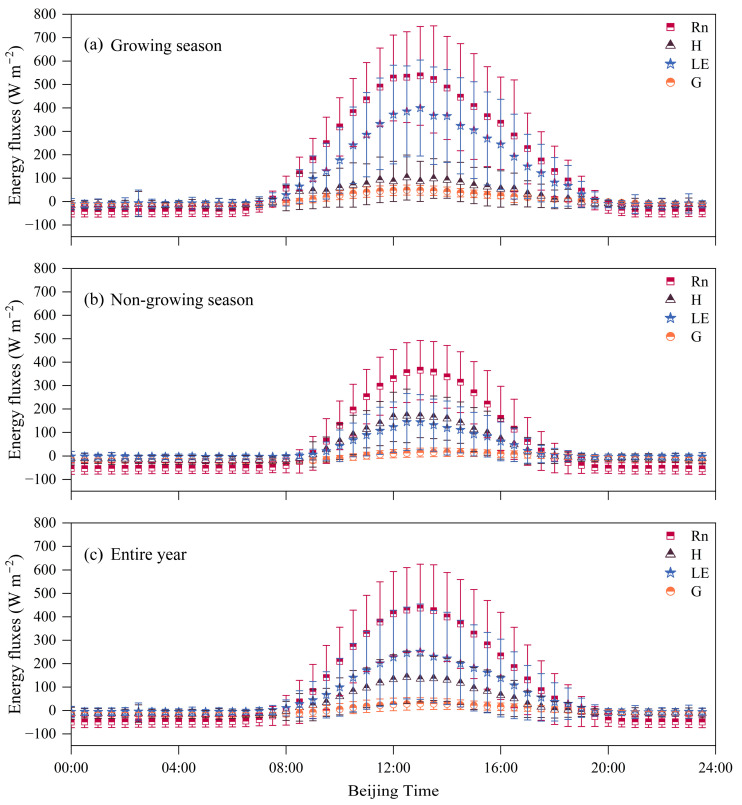
Daily variation in energy fluxes during the GS (**a**), NGS (**b**), and throughout the year (**c**) at the meteorological observation station in 2022–2023.

A paired *t*-test was performed on the half-hourly data of *R_n_*, *G*, *H*, and *LE* during the GS and NGS. The results indicate significant differences in surface fluxes between the GS and NGS (** p < 0.01). Figure 2 illustrates the daily variation in energy fluxes in vegetation during the GS, NGS, and throughout the year. The daily variation trends of *R_n_*, *H*, *LE*, and *G* display an n-shape, with *R_n_* exhibiting the most pronounced and intense changes. The maximum peak typically occurs at 13:00, with an annual average of 437.4 W m^−2^. The peak during the GS is 536.94 W m^−2^, significantly higher than that of the NGS at 365.56 W m^−2^. Compared to other fluxes, the variation magnitude follows the order of *LE* > *H* > *G*, with the timing of the *G* peak being inconsistent. In the NGS, the peak occurs later, usually at 13:30 (15.95 W m^−2^), while during the GS, the peak is reached earlier at 12:30, being three times that of the NGS at 49.14 W m^−2^. The annual peak occurs at 13:00 (28.89 W m^−2^). Throughout the year, among the turbulent fluxes (*H* and *LE*), the overall average of *LE* is higher than that of *H*, and the timing of their peaks also differs. The annual daily average peak of *H* occurs at 12:30, measuring 142.27 W m^−2^, which is lower than the peak of *LE* (196.58 W m^−2^) and occurs half an hour earlier. During the GS, the peak of *H* occurs at 12:30 (102.67 W m^−2^), while the peak of *LE* occurs later at 13:00 (399.46 W m^−2^). In the NGS, the peak of *H* exceeds that of *LE*, both occurring at 12:30, measuring 170.84 W m^−2^ and 144.47 W m^−2^, respectively.

### 2.3. Seasonal Distribution and Proportional Characteristics of Energy Fluxes

**Figure 3:**
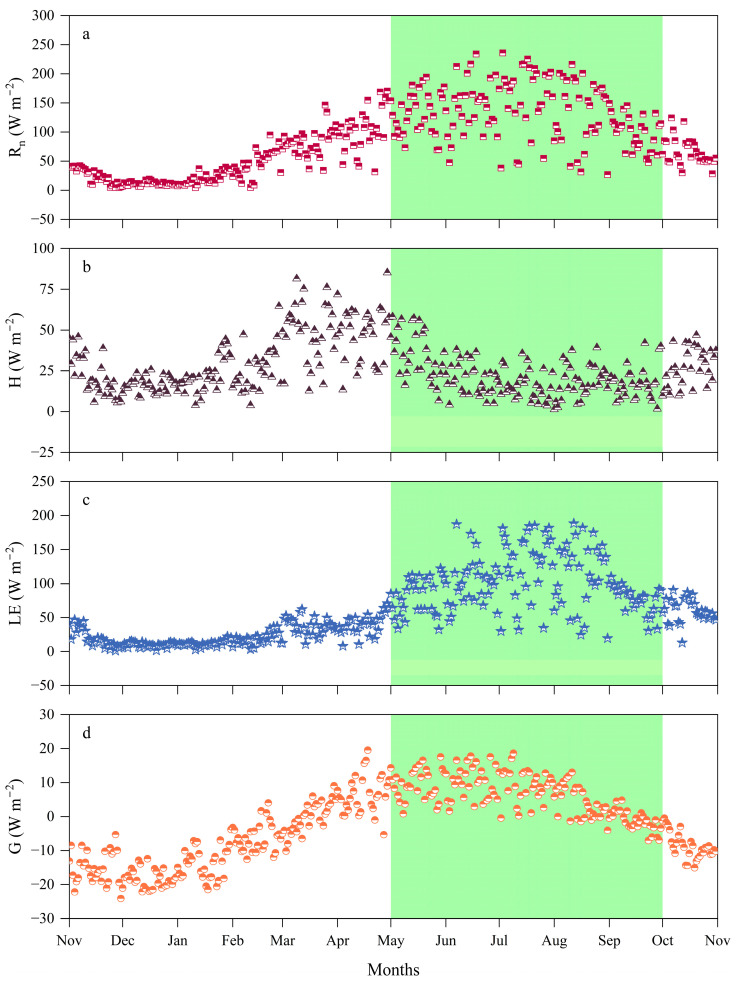
Yearly variation in daily average energy fluxes at the meteorological observation site from 2022 to 2023, with the green area indicating the GS: (**a**) *R_n_*; (**b**) *H*; (**c**) *LE*; (**d**) *G*.

**Figure 4:**
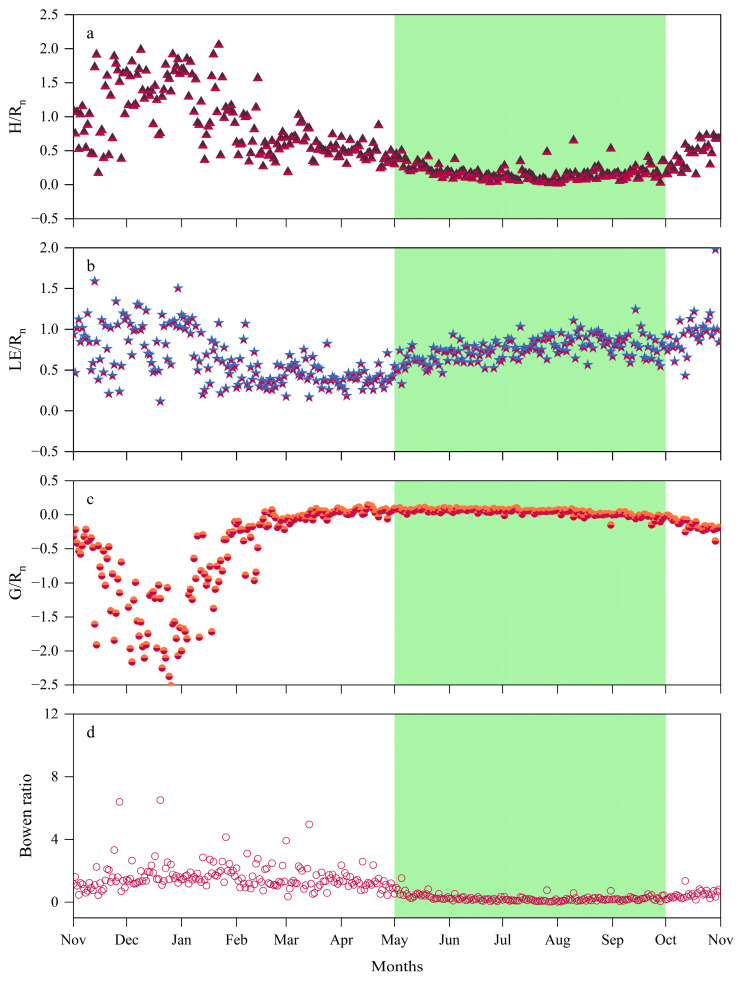
Yearly variations in the daily average proportions of energy fluxes and Bowen ratio at the meteorological observation site from 2022 to 2023, with the green area indicating the GS: (**a**) *H/R_n_* ratio; (**b**) *LE/R_n_* ratio; (**c**) *G/R_n_* ratio; (**d**) Bowen ratio.

**Figure 5:**
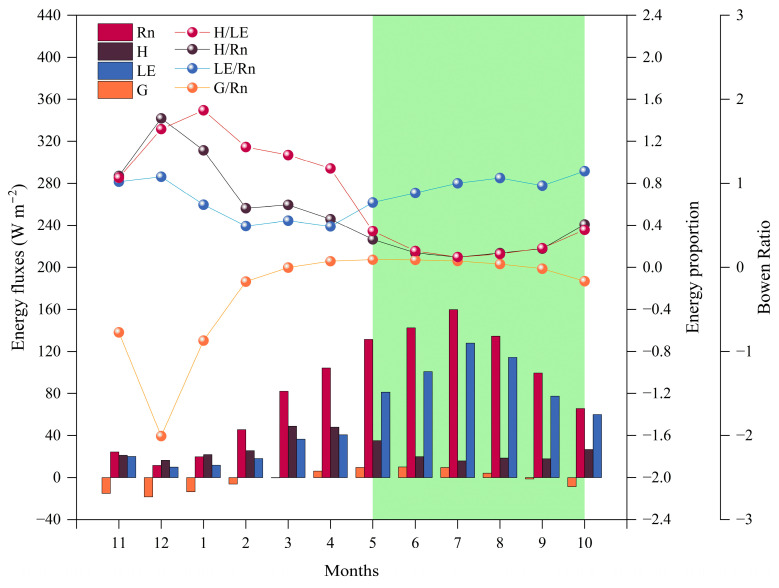
Yearly variations in the monthly average proportions of energy fluxes and Bowen ratio at the meteorological observation site from 2022 to 2023, with the green area indicating the GS.

Figure 3, Figure 4 and Figure 5 illustrate the trends and distribution characteristics of daily and monthly average values of surface energy components (*R_n_*, *H*, *LE*, and *G*) in alpine meadows during the monitoring period, with the GS, NGS, and annual daily averages presented in Table 1. During the NGS, the daily average of *R_n_* is relatively low due to the lower solar radiation angle and shorter daylight hours. In December, the monthly average of *R_n_* reaches its minimum value of 11.46 W m^−2^, after which, *R_n_* steadily increases. Once the GS begins, with longer daylight hours, the daily average substantially exceeds that of the NGS, peaking at 159.81 W m^−2^ in July, followed by a gradual decline.

The seasonal variation in *LE* follows a similar trend to *R_n_* but the variations in *H* and *LE* differ substantially. In December, due to the lowest *R_n_* and the lowest temperatures of the year, surface evaporation nearly halts, and the monthly averages of *LE* and *H* drop to 9.88 W m^−2^ and 15.26 W m^−2^, respectively. However, at this point, the *H/R_n_* ratio exceeds the *LE/R_n_* ratio. In January, the Bowen ratio reaches its highest value, with the *R_n_* provided by solar radiation mainly converting to *H*. In March, the monthly average of *H* first reaches its peak (48.76 W m^−2^). By May, *R_n_* undergoes significant changes, and the monthly average of *LE* exceeds that of *H* for the first time, causing the Bowen ratio to drop below 1. This indicates that, with increasing *R_n_*, about two-thirds of the energy is converted into *LE*, while only one-third is transferred to the atmosphere as *H*. After entering the GS, with rapid vegetation growth and soil thawing, temperatures rise, and evaporation increases. *LE* peaks at 127.93 W m^−2^ in July, and then begins to decrease after August as *R_n_* gradually declines. During this period, the *LE/R_n_* ratio remains higher than the *H/R_n_* ratio, and the Bowen ratio stays below 1, indicating that evaporation from vegetation and soil plays a dominant role in the energy distribution of *R_n_*. Most of the *R_n_* returns to the atmosphere in the form of *LE*. During this period, *H* decreases and stabilizes, with a slight increase in October. The seasonal variation in *G* is relatively stable, often negative during the NGS, indicating that soil heat is primarily transferred to the colder air. In December, the monthly average of *G* reaches its lowest value of the year (−18.41 W m^−2^). After that, *G* gradually increases and becomes positive in March. Entering the GS, *G* continues to rise and peaks in June at a monthly average of 10.02 W m^−2^, when the soil absorbs the continuously increasing *R_n_*, and the *G/R_n_* ratio reaches its highest value. Surface soil has slower thermal conductivity and limited heat exchange, so the *G/R_n_* ratio remains consistently lower than both the *H/R_n_* and *LE/R_n_* ratios.

### 2.4. Factors Influencing Energy Fluxes

**Figure 6:**
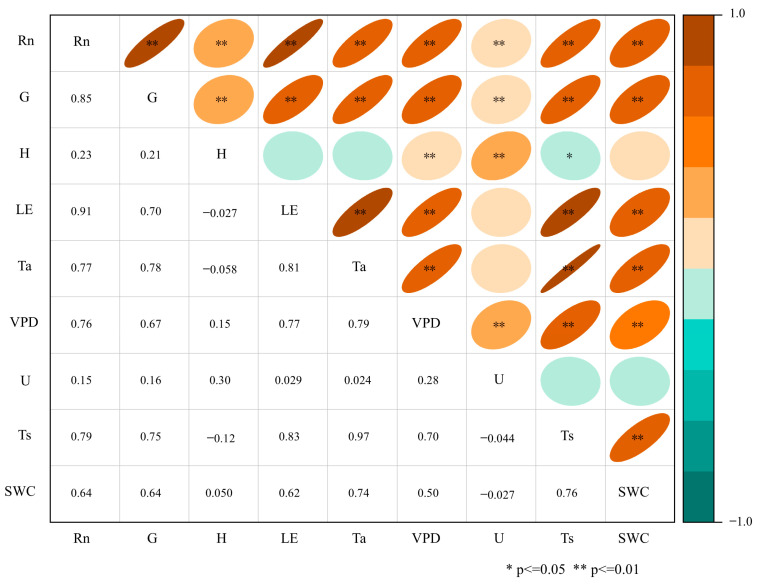
Pearson’s correlation coefficients between meteorological variables and energy fluxes during the study period.

**Figure 7:**
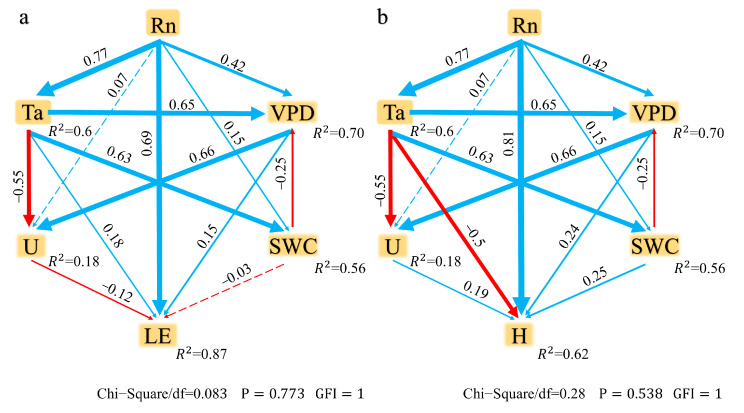
Structural equation model (SEM) of meteorological variables (*T_a_*, *VPD*, *U*, and *SWC*) and energy components (*R_n_*, *H*, and *LE*) at the observation site. Panels (**a**,**b**) represent the path analysis models for *LE* and *H*, respectively, with standardized path coefficients positively correlated with the strength of path arrows. Blue arrows indicate positive correlations, while red arrows represent negative correlations.

Figure 6 explores the relationship between meteorological variables (*R_n_*, *T_a_*, *VPD*, *U*, *T*_s_, and *SWC*) and energy fluxes (*H* and *LE*) through Pearson’s correlation analysis. Based on the correlation results, a path analysis model was constructed in Figure 7 to analyze the direct and indirect effects of meteorological variables on *H* and *LE*.

The results of Pearson’s correlation analysis show that *R_n_* is significantly positively correlated with both *H* and *LE*, indicating that *R_n_* is the primary driving factor for both. The correlation between *T_a_* and *H* is weak, indicating that changes in *T_a_* have a less significant impact on *H* compared to *LE*. *VPD* is positively correlated with both *H* and *LE*, suggesting that under dry conditions, an increase in *VPD* enhances the flux of both. The correlation between *U* and *H* is small, while its correlation with *LE* is weak, indicating that *U* has a limited effect on energy fluxes. *SWC* shows a high positive correlation with *LE* but a lower correlation with *H*, indicating that wet soil conditions are more conducive to an increase in *LE*. Based on the results of Pearson’s correlation analysis, path analysis models for *H* and *LE* were constructed. During the model construction process, variables with higher correlation coefficients were prioritized as main pathways. *R_n_* exhibited high correlations in both models, thus serving as the core driving factor in the path models. In the *H* model, the strong correlation between *T_a_* and *H* led to *T_a_* being included as a direct influencing factor. Although *VPD* has a relatively low correlation with *H*, its impact on air dryness allows it to be included as an indirect influencing variable in the model. *U* and *SWC*, having lower correlations with *H*, were given lower priority in the model. In the *LE* model, *SWC* shows the strongest correlation with *LE*, leading to its prioritization for inclusion, while *U* was also considered due to its promoting effect on evaporation and transpiration processes.

In the path analysis model for *H*, *R_n_* has a significant direct effect on *H*, with a path coefficient of 0.81, indicating that *R_n_* is the main driving factor for *H*. The path coefficient for *T_a_* on *H* is −0.50, indicating a negative correlation between rising temperatures and *H*, suggesting that high-temperature conditions may suppress *H*. The path coefficient for *VPD* on *H* is 0.24, showing that in dry environments, the increase in *VPD* contributes relatively little to *H*. The path coefficient for *U* is 0.19, indicating that its effect on *H* is relatively small, yet it still contributes to energy exchange. The path coefficient for *SWC* is 0.25, indicating that *H* increases under moist soil conditions.

*R_n_* also has a significant effect on *LE*, with a path coefficient of 0.69, emphasizing its core role in *LE*. The path coefficient for *SWC* on *LE* is −0.03, which is weak and not significant, while *T_a_* has a path coefficient of 0.18, indicating a positive but limited effect on *LE*. The path coefficient for *VPD* on *LE* is 0.15, suggesting that an increase in *VPD* can promote the enhancement of *LE*. The path coefficient for *U* is −0.12, suggesting that an increase in *U* may have a negative impact on *LE*. The analysis results from the structural equation model clearly reveal the complex relationships of meteorological factors in the transmission of energy fluxes.

## 3. Discussion

### 3.1. Seasonal Variations in Energy Components

The three energy components, *H*, *LE*, and *G*, exhibit significant seasonal variations influenced by climate change and underlying surface conditions during the growth of vegetation in alpine meadows [42]. Comparing the results of this study with other research on alpine meadows in the QTP, it was found that the seasonal variation trends of surface energy fluxes (*R_n_*, *G*, *H*, and *LE*) are consistent across different growth periods; however, differences in daily averages were observed. Compared to the western QTP regions of Xainza and Maqin, the daily averages of *R_n_* and *H* are lower in this study area, with *G* values being similar, while *LE* is relatively higher [43,44]. In comparison to the central QTP regions of Zhiduo and Wudaoliang, *R_n_* and *LE* are similar but *G* and *H* are relatively lower [45,46]. When compared with the eastern Qilian Mountains, particularly the Tianlaochi region, *G* and *H* are lower, while *LE* is higher, with *R_n_* being significantly smaller [4]. The differences in *R_n_* are likely influenced by factors such as elevation, topography, solar angles, and clear weather conditions [5,47]. The QTP region, with an elevation typically exceeding 4000 m, leads to higher *R_n_* intensities at high altitudes. In the Tianlaochi region of the Qilian Mountains, *R_n_* is relatively higher at the same elevation, while in the Arou region, *R_n_* is similar [18]. The energy variation processes in our study area are weaker than in the QTP but similar to those in the Qilian Mountains. The Qilian Mountains are influenced by the monsoon earlier, resulting in a longer and more vigorous growing season. This causes an earlier shift in surface energy from *H* to *LE* [48,49].

The energy distribution during different growth periods is closely related to the state of meadow vegetation. During different time periods, either *H* or *LE* dominates the energy allocation. The distribution of *LE* and *H* in *R_n_* significantly impacts local climate and hydrological processes [50]. The Bowen ratio (*β*, *H/LE*) serves as a key indicator of surface energy distribution [51]. During the NGS, as Rn decreases, *H*, *LE*, and *G* also decrease. Under conditions of snowfall and dry, cold temperatures, most of *R_n_* is released to the atmosphere via *H*, making *H* the dominant energy flux and leading to *β* > 1. During this period, when the soil freezes, *G* typically has negative values, indicating that heat is being transferred from the soil to the cold air. Toward the end of the NGS, as *R_n_* and *T_a_* increase, *H* reaches its first peak in March–April, while the meadow begins to sprout and grow, leading to increases in *LE* and *G*. During the GS, *R_n_* peaks in July, and *G* and *LE* reach their peaks slightly later in the month, trailing behind *H*. Currently, energy mainly returns to the atmosphere via evaporation from vegetation leaves and soil moisture, resulting in an increase in *LE* and a decrease in *H*, leading to *β* < 1. The soil absorbs a significant amount of heat from the atmosphere, and this heat reaches its peak during this period [52]. However, as the GS transitions into the NGS, *R_n_* and *T_a_* decrease, causing vegetation to wilt. As a result, transpiration declines, *LE* decreases, and *H* reaches a second minor peak. This regular variation in the surface energy components during vegetation growth in alpine meadows is also observed in other regions of the QTP [4,15,19,31,53].

### 3.2. Factors Influencing Energy Fluxes in Alpine Meadow Ecosystems

The path analysis model used in this study indicates that the primary factor influencing the surface turbulent flux in alpine meadows is *R_n_*, which shows a significant positive correlation with surface turbulent fluxes. This relationship has been observed in numerous studies [18,54,55]. In addition to *R*_n_, *T_a_* is also a crucial factor affecting surface turbulence. The continuous increase in *R_n_* requires a corresponding rise in *T_a_* to maintain energy balance at the surface [56]. From the NGS to the GS, the increase in *R_n_* causes a rise in *T_a_*, leading to soil thawing, changes in soil bulk density, and a sharp increase in *SWC*, which in turn increases *LE* [57,58,59]. As the vegetation recovers from dormancy, the atmospheric humidity and VPD increase, further enhancing evaporation rates [60]. With vegetation growth, the leaf area expands, altering surface albedo and roughness, which further affects the distribution of turbulent fluxes through transpiration [59]. In April, the weakening of *U* balances the temperature difference, while the sharp rise in SWC promotes root water absorption, fostering plant growth. Increased photosynthesis and respiration enhance the absorption of *R_n_* and its conversion to *LE*, leading to a decline in *H* and an increase in *LE* during this phase [45]. In the mid-GS, when *T_a_* peaks, transpiration rates accelerate, causing *LE* to reach its maximum [61,62]. At this point, the higher vegetation coverage reduces albedo and inhibits the soil temperature rise, leading to a decrease in *H* [63]. The intense evaporation processes from vegetation and the moist soil surface contribute to the peak in *LE* [64]. The increase in *LE* corresponds to climate warming and reflects the growing phenomenon of shrubification in the alpine meadows at the study site. Excessively high *T_a_*, along with *VPD*, can cause plants to close their stomata as a self-protective measure, thus reducing photosynthesis and transpiration rates [65]. An increase in *SWC* also limits root absorption of moisture, further decreasing evaporation rates [66]. The longer duration of *LE* dominance during the GS shortens the soil freezing and thawing period, and an increased *SWC* leads to more extensive melting of the frozen soil layer, releasing more heavy metals, which contribute to meadow degradation [67]. The impact of precipitation at different stages on surface turbulence is also noteworthy. During the NGS, precipitation is limited but snowfalls are frequent. The melting of snow and the formation of new snow temporarily affect *LE*, leading to its increase. The high albedo of accumulated snow reflects more *L_u_*, thereby reducing *R_n_* [68]. During this snowmelt process, significant and unstable daily fluctuations in the *H/R_n_* and *LE/R_n_* ratios are observed in the NGS. In contrast, during the GS, precipitation is primarily in the form of rainfall. During the rainy season, *SWC* is generally higher than in the dry season [69]. Rainfall increases the water vapor content in the atmosphere, which enhances plant growth and leads to an increase in evapotranspiration, causing *LE* to peak [35,70]. In the GS, vegetation gradually increases its leaf area, continuously changing surface albedo and roughness, which further affect the distribution of turbulent fluxes through transpiration [59]. During the early GS, the expanding leaf area of vegetation reduces the soil reflection of Rn, which in turn reduces the temperature difference between the atmosphere and surface, lowering *H* [63]. In the mid-GS, the combined processes of intense photosynthesis, respiration, and soil evaporation from moist surfaces drive *LE* to its peak [64].

Nevertheless, this study has certain limitations. During surface flux studies in alpine meadows, an energy imbalance is often observed [28]. The BREB, based on the *K_h_* = *K_v_* assumption, is a simplified model for studying surface flux variations and requires strict meteorological conditions. The accuracy of the experimental instruments, atmospheric instability, and the use of roughness parameters derived from similar regions also impact the results, leading to an energy imbalance. Additionally, other influencing factors, such as atmospheric cloud cover variations during different growth periods, were not considered and may affect direct solar radiation reaching the surface. The study area has a long snow-covered period, and a lack of records on snowmelt reduces the accuracy of *LE* calculations during the NGS. The absence of surface albedo references for different seasons, along with the lack of records for the vegetation leaf area index or specific dates of growth and dormancy, also affects the study of surface flux variations in alpine meadows.

## 4. Materials and Methods

### 4.1. Study Site

**Figure 8:**
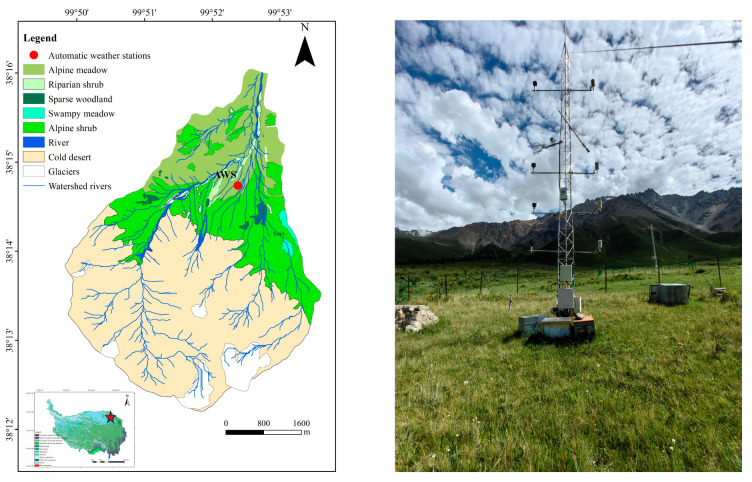
Location of the Hulu catchment (red dot) and meteorological observation stations.

This study was conducted in the Hulu catchment of the upper Heihe River basin in the QTP (latitude 38°12′–38°17′, longitude 99°50′–99°54′; see Figure 8) [71]. The elevation of the catchment ranges from 2960 to 4820 m, covering an area of 23.1 km^2^ [72]. The catchment exhibits distinct alpine mountain climatic characteristics, with a climate type classified as continental and cold humid. Temperatures are low and highly variable, with precipitation occurring concurrently with temperature changes. The annual average daily temperature and precipitation recorded by the National Meteorological Station from 2022 to 2023 were 0.86 °C and 436.02 mm, respectively. Precipitation is primarily concentrated in the warm and humid growing season (from May to September), accounting for 88% of the annual total, while the remaining months (from October to April) are characterized by dry and cold conditions. The main vegetation and corresponding soil types in the catchment include the following: the alpine meadow community was dominated by *Carex myosuroides* Vill., with the surface soil classified as Mollisols. The riparian shrub community was characterized by *Hippophae tibetana* Schltdl., with the underlying soil classified as Inceptisols. The sparse woodland community consisted of *Picea crassifolia* Kom. and *Juniperus przewalskii* Kom., with soils primarily classified as Inceptisols and Mollisols. The swampy meadow community was dominated by *Blysmus compressus* (L.) Panz. Ex Link, with the soil classified as Histosols. The alpine shrub community had species such as *Dasiphora fruticosa* (L.) Rydb. and *Caragana jubata* (Pall.) Poir., where the soils are a combination of Inceptisols and Entisols. The cold desert community was characterized by *Rhodiola rosea* L., with Gelisols as the predominant soil type [36].

### 4.2. Instruments and Measurements

We selected the meteorological observation station in the central alpine meadow of the Hulu catchment (38°24.9′ N, 99°87.4′ E), at an elevation of 3232 m. The observation area is a flat, open valley bottom, approximately 400 m from the mountain, with a slope of less than 10°. A 10 m × 10 m fenced area was established around the station to ensure the safety and stable operation of the equipment. Vegetation information at the study site was determined using sampling methods. The dominant vegetation was *K. myosuroides*, with the accompanying plant *C. moorcroftii*. During the GS (May to September), the vegetation cover is approximately 92%, with an average height of about 0.25 m. In the NGS (October to April of the following year), the average height of the vegetation is about 0.15 m.

An integrated environmental monitoring system was installed at the experimental site, including a four-component radiation sensor and a soil temperature sensor to collect data on different solar radiation components and surface air temperature. Air temperature and humidity sensors, as well as a wind speed sensor, were installed at two different heights. Soil sensors were placed at various soil depths, including soil temperature sensors, soil moisture sensors, and soil heat flux sensors. Detailed information on all sensors is provided in Table 2. All measured data were averaged over 30-minute intervals and stored using a data logger (CR1000, Campbell Scientific, Logan, UT, USA) set up at the experimental station. The system collected observational data from 1 November 2022 to 31 October 2023.

### 4.3. Bowen Ratio Energy Balance (BREB) Method

In this study, we used the BREB method to calculate surface energy changes. This method is well-established, reliable, and widely applied in numerous studies of surface energy variations [43]. According to the first law of thermodynamics, solar radiation entering the surface is absorbed and reflected. Equation (1) represents the mathematical formulation for surface energy balance:

(1) $$R_{n}=G+H+LE+S$$

Equation (2) calculates *R_n_* using data collected by the radiation instruments set up in the integrated environmental monitoring system:

(2) $$R_{n}=S_{d}-S_{u}+L_{d}-L_{u}$$

Equation (3) calculates *G* using embedded soil heat flux plates and temperature gradients at different soil depths:

(3) $$G=G_{P}+C_{s}∆z\frac{\partial T}{\partial t}$$

where *S* represents the energy produced by leaf metabolism and storage (W·m^−2^), which are usually neglected in calculations. In 1926, the Bowen ratio was proposed as the ratio of *H* to *LE*, assuming that the exchange coefficients for water vapor and heat between two horizontal planes perpendicular to the evaporation direction are similar, i.e., *K_h_* = *K_v_*. The corresponding mathematical equation is presented in Equation (4), outlined below:

(4) $$\beta =\frac{H}{LE}=\frac{\rho_{a}C_{p}K_{h}\left[T_{2}-T_{1}+\tau (Z_{2}-Z_{1})\right]}{\rho_{a}C_{p}K_{w}(e_{2}-e_{1})/\gamma }=\gamma \frac{T_{2}-T_{1}+\tau (Z_{2}-Z_{1})}{e_{2}-e_{1}}$$

To reduce errors, Foken et al. (2008) recommended that temperature and humidity be measured at two heights, with a height ratio of 4–8 [73]. In this study, *Z*_1_ and *Z*_2_ were chosen as 0.15 m and 7.70 m, respectively. Using Equations (1) and (4), we can calculate the *H* and *LE* as follows:

(5) $$LE=\frac{Rn-G}{\left(1+\beta \right)}$$

(6) $$H=\frac{\beta (Rn-G)}{\left(1+\beta \right)}$$

All abbreviations are shown in Table 3.

### 4.4. Exclusion of Outliers and Imputation

The results of the BREB model assume that *K_h_* = *K_v_*, which requires the underlying surface to be homogeneous. According to the turbulent flux footprint model proposed by Hsieh et al. (2000) [74], the 90% fetch-to-measurement height ratio is 54 m. Considering that the heights of the two humidity and temperature sensors are 1.5 m and 7.7 m, respectively, their geometric mean is 3.4 m. This indicates that 90% of the effective turbulent flux is confined within a radius of 184 m from the meteorological tower. Meanwhile, the homogeneity of the experimental area is sufficiently stable, making the results obtained from the BREB model reasonable.

To obtain continuously recorded data, this study utilized linear interpolation and the average diurnal variation method to fill in missing meteorological data caused by extreme weather and instrument failures [75]. After data processing and imputation of missing values, the data were further aggregated into daily averages. When using the BREB method to calculate surface fluxes, turbulence data obtained during the morning and evening are often unreliable.

To ensure the reliability of the results obtained using the BREB method, quality control was implemented based on the dynamic error exclusion standard (β) proposed by Hu et al. (2014), which is based on the typical instrument resolution. The following quality control criterion was applied to the computed data [76]:

(7) $$-1-\left|\frac{\delta_{1}-{\gamma \delta }_{2}}{∆e}\right|<\beta <-1+\left|\frac{\delta_{1}-{\gamma \delta }_{2}}{∆e}\right|$$

This screening process ensures the accuracy of the turbulent flux calculations. For missing data in the BREB method, the flux profile method was used for calculation and imputation [45].

### 4.5. Energy Closure Calculation

Due to the prevalent energy imbalance in alpine meadows [19,77], it is necessary to verify the reliability of daily averaged energy values on the surface. The energy closure ratio (EBR) typically ranges from 0.55 to 0.99. To validate this, we used the energy closure calculation formula (Equation (8)) and performed linear regression between the available energy and turbulent flux:

(8) $$EBR=\frac{\sum \left(H+LE\right)}{\sum \left(R_{n}-G\right)}$$

**Figure 9:**
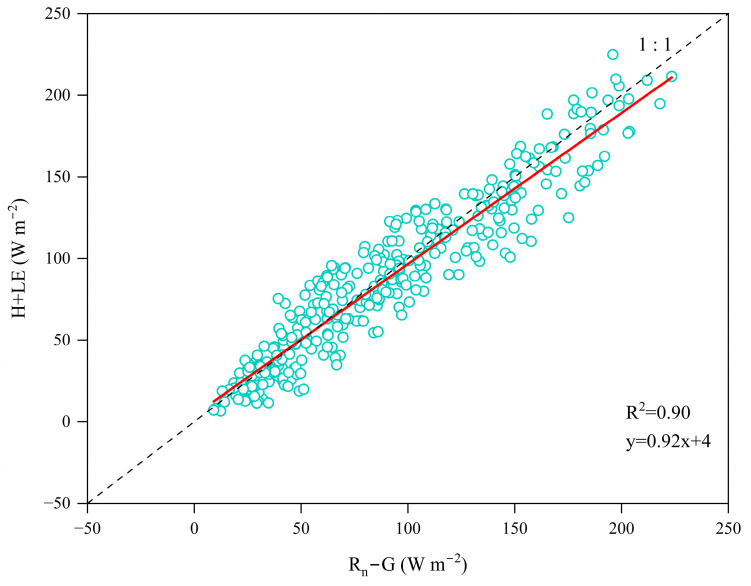
Linear correlation analysis between daily average turbulent flux (*H* + *LE*) and available flux (*R_n_* + *G*).

The energy balance closure ratio (EBR) was calculated to be 0.85. As shown in Figure 9, the slope of the linear regression was 0.92, indicating a strong correlation between turbulent flux and available energy. This suggests that the calculated turbulence data are reliable [18,78].

### 4.6. Data Analysis

The surface fluxes of *R_n_*, *G*, *H*, and *LE* exhibit significant differences between the GS and NGS. To assess these differences, a paired *t*-test was conducted on the half-hourly data. To determine the relationship between *H* and *LE* and their potential influencing factors (*T_a_*, *T_s_*, *U*, *VPD*, *R_n_*, *SWC*), we conducted Pearson’s correlation analysis to assess the strength and direction of linear relationships between these variables. Simultaneously, a Pearson correlation heatmap was generated using Origin software (OriginLab Corporation, Northampton, MA, USA), version 2024. The heatmap visually represents the correlation coefficients between variables, with * p < 0.05 and ** p < 0.01 indicating significant and highly significant relationships, respectively (see Figure 6). Based on the results of Pearson’s correlation analysis, path analysis models for *H* and *LE* were constructed. Path analysis allowed us to quantify the direct and indirect effects of the meteorological factors on *H* and *LE*, providing a comprehensive understanding of the energy flux dynamics. We utilized the Goodness of Fit Index (GFI) and Chi-Square/df ratio to evaluate the performance and adequacy of the path analysis model. The model was considered well-constructed if the Goodness of Fit Index (GFI) was greater than 0.9 and the Chi-Square/df ratio was less than 3. All statistical analyses were conducted using the SPSS Amos 22.0 platform (IBM Inc., Armonk, NY, USA) [79].

## 5. Conclusions

This study collected meteorological data during the growth process of an alpine meadow and applied the BREB model to calculate the surface energy balance. This study focuses on analyzing the variations, proportions, and influencing factors of surface fluxes during different time periods. The results show that the daily peak values of surface fluxes occur around 1:00 PM, with lower values in the growing season (GS) compared to the non-growing season (NGS). Although the trends are consistent, there are significant differences in the magnitudes of the variations. On an annual scale, the LE/Rn ratio is higher than the H/Rn ratio, and G occupies the smallest proportion throughout the year, with most Rn being returned to the atmosphere as LE. Specifically, in the growing season, Rn is primarily converted to H, while in the non-growing season, it is mainly converted to LE. The path analysis and Pearson’s correlation analysis both indicate that Rn is the primary driver of both LE and H, followed by Ta, which has a stronger effect on H than on LE. VPD and SWC have a stronger impact on LE than on H, while U has a limited influence on surface fluxes. Overall, this study provides foundational data on the climate response of alpine meadows; however, the observation period was relatively short, and future research could benefit from using remote-sensing data to track long-term vegetation growth changes over a wider area, with an emphasis on the impact of extreme weather on the energy balance and the feedback mechanisms between vegetation growth and energy fluxes.

## Figures and Tables

**Table 1:** Average values of energy composition and major meteorological factors in alpine meadow ecosystems during different periods.

Item	Growing Season (May–September)	Non-Growing Season (October–April)	Entire Year
*T_a_* (°C)	7.12	−7.77	−1.53
*T_s_* (°C)	6.41	−6.89	−1.31
*VPD* (kP_a_)	0.47	0.24	0.33
*U* (m s^−1^)	2.19	2.24	2.22
*SWC* (m^3^ m^−3^)	0.32	0.16	0.23
*R_n_* (W m^−2^)	133.70	50.35	85.29
*H* (W m^−2^)	21.36	29.79	26.25
*LE* (W m^−2^)	100.47	28.22	58.50
*G* (W m^−2^)	6.41	−7.99	−1.95
*H/R_n_*	0.17	0.84	0.56
*LE/R_n_*	0.75	0.67	0.71
G/*R_n_*	0.04	−0.57	−0.32
*β*	0.25	1.42	0.93

**Table 2:** List of meteorological station instruments and installation information.

Variable	Sensor	Manufacturer	Accuracy	Height (m)
Net radiation (*R_n_*)	CNR1	Kipp & Zonen Kipp & Zonen (Delft, The Netherlands)	±1%	1.5
Air temperature (*T_a_*)	41382VC; HMP 155A	R. M. Young (Traverse City, MI, USA); Vaisala (Helsinki, Finland)	±0.05 and ±0.2 °C	1.5, 7.7
Relative humidity (*RH*)	41382VC; HMP 155A	R. M. Young (Traverse City, MI, USA); Vaisala (Helsinki, Finland)	±1 and ±2%	1.5, 7.7
Wind speed and direction (*U*)	Wind Sonic; Young05103	Gill (Lymington, Hampshire, UK); R.M. Young (Traverse City, MI, USA)	±0.01 and ±0.3 m·s^−1^	1.5, 7.7
Surface temperature (*T_s_*)	SI-111	Apogee (Logan, UT, USA)	±0.2 °C	1.5
Soil heat flux (*G*)	HFP01SC	Hukseflux(Delft, The Netherlands)	±3%	−0.05 −0.20
Soil water content (*SWC*)	Enviro SMART	Sentek (Adelaide, Australia)	±0.1%	−0.05 −0.20
Soil temperature (*T_S_*_5_, *T_S_*_20_)	109SS-L	Campbell Scientific (Logan, UT, USA)	±0.2 °C	−0.05 −0.20

**Table 3:** Abbreviation list for the BREB method.

*R_n_*	Net radiation, W m^−2^	*K_h_*	Heat exchange coefficient
*S_d_*	Downward shortwave radiation, W m^−2^	*K_v_*	Water vapor exchange coefficient
*S_u_*	Upward shortwave radiation, W m^−2^	*T*_1_	Air temperature at Z_1_, K
*L_d_*	Downward longwave radiation, W m^−2^	*T*_2_	Air temperature at Z_2_, K
*L_u_*	Upward longwave radiation, W m^−2^	*e*_1_	Vapor pressure at Z_1_, kP_a_
*G*	Soil heat flux, W m^−2^	*e*_2_	Vapor pressure at Z_2_, kP_a_
Δ*z*	Soil layer thickness, m	*ρ_a_*	Mean air density at constant pressure, kg m^−3^
*G_p_*	Energy measured by the embedded soil heat flux plates, W m^−2^	*C_p_*	Specific heat capacity of air at constant pressure, J kg^−1^ K^−1^
*C_s_*	The volumetric heat capacity of the soil layer (J m^−3^ K^−1^)	*τ*	Adiabatic lapse rate, which is normally taken as 0.01 K m^−1^
*β*	Bowen ratio	Δ*e*	Vapor pressure difference between the lower and the upper measurement levels, kP_a_
*∂T/∂t*	Rate of variation in the mean temperature of the soil layer	*SWC*	Soil water content at a depth of 5 cm, m^3^ m^−3^
*T_a_*	Air temperature at a height of 2 m above the ground, °C	*VPD*	Vapor pressure deficit, kP_a_
*U*	Wind speed at a height of 2 m above the ground, m s^−1^	*BREB*	Bowen ratio energy balance
*T_s_*	Soil temperature at a depth of 5 cm, °C	*GS*	Growing season
*H*	Sensible heat flux, W m^−2^	*NGS*	Non-growing season
*LE*	Latent heat flux, W m^−2^	*δ*_1_	The absolute error limit of vapor pressure difference Δ*e*
*γ*	Psychorometric constant, (kPa K^−1^)	*δ*_2_	The absolute error limit of vapor pressure difference Δ*T*

## Data Availability

Data will be made available on request.
